# Where Do Female Sex Workers Seek HIV and Reproductive Health Care and What Motivates These Choices? A Survey in 4 Cities in India, Kenya, Mozambique and South Africa

**DOI:** 10.1371/journal.pone.0160730

**Published:** 2016-08-05

**Authors:** Yves Lafort, Ross Greener, Anuradha Roy, Letitia Greener, Wilkister Ombidi, Faustino Lessitala, Hassan Haghparast-Bidgoli, Mags Beksinska, Peter Gichangi, Sushena Reza-Paul, Jenni A. Smit, Matthew Chersich, Wim Delva

**Affiliations:** 1 International Centre for Reproductive Health, Ghent University, Gent, Belgium; 2 MatCH Research (Maternal, Adolescent and Child Health Research) Unit, University of the Witwatersrand, Durban, South Africa; 3 Ashodaya Samithi, Mysore, India; 4 International Centre for Reproductive Health-Kenya, Mombasa, Kenya; 5 International Centre for Reproductive Health-Mozambique, Maputo, Mozambique; 6 Institute for Global Health, University College London, London, United Kingdom; 7 University of Nairobi, Nairobi, Kenya; 8 University of Manitoba, Winnipeg, Canada; 9 Discipline of Pharmaceutical Sciences, College of Health Sciences, University of KwaZulu-Natal, Durban, South Africa; 10 Wits Reproductive Health and HIV Institute, Faculty of Health Sciences, University of the Witwatersrand, Johannesburg, South Africa; 11 The South African DST/NRF Centre of Excellence in Epidemiological Modelling and Analysis (SACEMA), University of Stellenbosch, Stellenbosch, South Africa; 12 Center for Statistics, Hasselt University, Diepenbeek, Belgium; 13 Rega Institute for Medical Research, KU Leuven, Leuven, Belgium; International AIDS Vaccine Initiative, UNITED STATES

## Abstract

**Background:**

A baseline cross-sectional survey among female sex workers (FSWs) was conducted in four cities within the context of an implementation research project aiming to improve FSWs’ access to HIV, and sexual and reproductive health (SRH) services. The survey measured where FSWs seek HIV/SRH care and what motivates their choice.

**Methods:**

Using respondent-driven sampling (RDS), FWSs were recruited in Durban, South Africa (n = 400), Tete, Mozambique (n = 308), Mombasa, Kenya (n = 400) and Mysore, India (n = 458) and interviewed. RDS-adjusted proportions were estimated by non-parametric bootstrapping, and compared across cities using post-hoc pairwise comparison tests.

**Results:**

Across cities, FSWs most commonly sought care for the majority of HIV/SRH services at public health facilities, most especially in Durban (ranging from 65% for condoms to 97% for HIV care). Services specifically targeting FSWs only had a high coverage in Mysore for STI care (89%) and HIV testing (79%). Private-for-profit clinics were important providers in Mombasa (ranging from 17% for STI care and HIV testing to 43% for HIV care), but not in the other cities. The most important reason for the choice of care provider in Durban and Mombasa was proximity, in Tete ‘where they always go’, and in Mysore cost of care. Where available, clinics specifically targeting FSWs were more often chosen because of shorter waiting times, perceived higher quality of care, more privacy and friendlier personnel.

**Conclusion:**

The place where care is sought for HIV/SRH services differs substantially between cities. Targeted services have limited coverage in the African cities compared to Mysore. Convenience appears more important for choosing the place of care than aspects of quality of care. The best model to improve access, linking targeted interventions with general health services, will need to be tailored to the specific context of each city.

## Introduction

Female sex workers’ (FSWs) access to HIV and other sexual and reproductive health (HIV/SRH) services and commodities is often hampered by fear of stigmatisation and discrimination at general health facilities, high mobility and lack of familiarity with the locally available services, inappropriate opening hours and the sometimes illegal immigration status [1–3]. Adequate and prompt care is nevertheless of utmost importance because FSWs are a key population in the fight against HIV and at markedly high risk for HIV, other sexually transmitted infections (STI) [4–6] and unintended pregnancies [7–9]. For these reasons, separate services are often established that specifically target FSWs, either through separate stand-alone clinics, drop-in centres (DICs) or community outreach [10]. The place where FSWs seek care for different HIV/SRH services and the motives for this choice have rarely been documented [2, 11] and little is known about what coverage targeted services achieve. In the context of an implementation research project that aims to improve the use of HIV/SRH services by FSWs, we therefore assessed at baseline where FSWs go for care in different settings, and what motivates this choice.

## Methods

The DIFFER (Diagonal Interventions to Fast-Forward Enhanced Reproductive Health) project is designed as a set of case studies, each in a well-defined geographical area, where sex work is common [12]. These are Durban, South-Africa; the Tete-Moatize area in Mozambique (hereafter referred to as ‘Tete’); Mombasa, Kenya; and Mysore, India. In each, HIV/SRH services are provided by public health facilities, private-for-profit clinics, as well as services targeted at FSWs. In Durban, targeted services are primarily provided through outreach by non-governmental organisations. In Tete, Mombasa and Mysore, there are in addition stand-alone clinics operated by non-governmental institutions. In Tete this is a small clinic at the outskirts of Moatize, called the Night Clinic [13], in Mombasa, three drop-in clinics in different divisions [14] and a clinic operated in the context of a research project [15], and in Mysore, a clinic operated by the FSW association Ashodaya Samithi [16]. At baseline, a detailed situational analysis was conducted in each city to inform the development of site and context-specific packages of interventions to strengthen HIV/SRH service delivery. This analysis included an assessment of where FSWs seek care for HIV/SRH, the reasons for the choice of the place of care, and the coverage of the targeted services.

FSWs (defined as women having received money or gifts for sex at least three times in the last six months) were recruited using Respondent-Driven-Sampling (RDS) in 2012–2013. RDS is similar to snowballing, but corrects for the bias towards FSWs with large social networks through statistical adjustments [17]. First, a limited number of known members of the FSW population are selected (seeds) who then are requested to invite other FSWs from their social circle for the survey. These in turn invite other FSWs, and so on. In Durban, 11 seeds were recruited, in Tete 13, in Mombasa 16, and in Mysore 8. In Durban, seeds were categorised according to age, indoor/outdoor SW and migration status, in Tete according to nationality (Mozambican/ Zimbabwean), place of residence (Tete city/ Moatize city) and type of FSW (full-time/ occasional), and in Mombasa according to location of soliciting sex (bar/club based, street/truck based, brothel/home based, and beach based). Each participant recruited up to three (Durban, Tete, Mombasa) or five (Mysore) new participants using coupons. Issuance and receipt of coupons was monitored in Durban, Tete and Mombasa using Electronic RDS Coupon Manager Version 3.0 and in Mysore manually through a coupon log notebook. To allow the detection of substantial changes in the main project indicators (namely the percentage of FSWs that uses contraception and the percentage of FSWs that received an HIV test in the previous 6 months) between the baseline and the end-of-project assessment, with a significance level of 0.05 and a power of 0.80, we estimated that a minimum sample size of 400 FSWs in each city was required. In Tete, recruitment was stopped after 6 months at 308 FSWs because of time constraints. Refusal rate was high in this city, in particular among FSWs of Mozambican nationality. In the other cities, the required sample was reached after less than 3 months. In Durban and Mombasa it was stopped because an equilibrium was achieved with respect to the composition of the categorized variables. In Mysore, recruitment was continued until the chains were completed and a total of 458 FSWs were enrolled.

FSWs were informed about the survey and gave their written consent to participate at a private and secure place. FSWs younger than 18 years were excluded. At all sites, consenting FSWs were interviewed face-to-face by a trained interviewer. In Durban, Mombasa and Mysore this was using a paper-based questionnaire and in Tete using Computer-Assisted Personal Interview software (QDS™). The questionnaire asked where the FSW usually obtains male condoms, where she normally goes for healthcare and, if applicable, where she last obtained the contraception method she uses, where she went the last time she had an abnormal vaginal discharge or genital ulcer, where she was last tested for HIV, where she is being followed for her HIV infection, and where the last cervical cancer test was done. Each time she was asked why she went there instead of somewhere else.

The study was approved by ethical boards in each country (the University of Witwatersrand’s Human Research Ethics Committee in South Africa, the National Committee of Bioethics for Health in Mozambique, the Kenyatta National Hospital/University of Nairobi Ethics and Research Committee in Kenya, and the Asha Kirana Institutional Ethics Committee in India), and by the Commission for Medical Ethics of the University Hospital Ghent in Belgium.

Questionnaires were entered in an MS-Access database in Durban, Mombasa and Mysore, and uploaded in a QDS data warehouse in Tete. The survey data were merged with the coupon data, and imported into STATA (Version 14, College Station, TX). In the analysis, we compared the place where care was sought for different HIV/SRH services and commodities across the four cities. We used the STATA RDS analysis package with the Volz-Heckathorn estimator (RDS II estimator) to calculate population point estimates adjusted for social network size and homophily within networks [18]. For the comparison among cities, we performed post-hoc pairwise comparison tests after fitting a logistic regression model with RDS-adjusted weights, using jack-knife resampling and Dunn–Šidák correction for multiple comparisons [19].

To assess the reasons for choosing the place of care and variations in reasons by type of service, we created an additional database with each care-seeking event for contraception, STI care, HIV testing services (HTS) or HIV care as a separate observation (6264 care seeking events by 1556 FSWs). Then, we compared the reasons for the choice of place of care across the cities and by type of place where care was sought, by fitting a multivariate logistic regression model with RDS-adjusted weights, using jack-knife resampling and adjusting for the cluster effect of care-seeking events by the same FSW. To control for confounding, both city and place of care were included in the model, as well as those FSWs’ socio-demographic characteristics that were associated with both the exposure and the outcome, and that altered odds ratios (ORs) by at least 10%.

## Results

### Socio-demographic characteristics

Table 1 summarises the sociodemographic and sex work characteristics of the interviewed FSWs. Participants’ characteristics differed between cities, in particular between Mysore and the African cities. Sex workers in the African cities were younger, better educated, more mobile, had more often a higher number of clients and less often a regular partner. Particular to Mozambique was that more than half of FSWs was of foreign origin, while in the other cities they were almost all nationals.

**Table 1:** Socio-demographic and sex work characteristics, by city.

Characteristic	Durban (N = 400)		Tete (N = 308)		Mombasa (N = 400)		Mysore (N = 458)	
	RDS-Adjusted		RDS-Adjusted		RDS-Adjusted		RDS-Adjusted	
	%	95% CI	%	95% CI	%	95% CI	%	95% CI
Age (years)								
Median	**27**		**29**		**26**		**34**	
< = 20	**6.4**	3.6–9.7	**15.6**	9.0–23.8	**11.6**	7.5–16.3	**0.3**	0.2–0.8
21–25	**37.3**	30.1–44.4	**20.6**	15.3–26.6	**30.6**	24.6–37.5	**16.6**	11.2–23.4
26–30	**31.3**	24.9–38.1	**27.1**	20.3–34.5	**29.0**	23.5–34.7	**33.0**	20.8–42.1
31–35	**12.8**	8.7–17.3	**19.8**	14.6–25.6	**15.7**	11.0–21.1	**19.5**	13.7–25.2
> = 36	**12.2**	6.7–18.4	**16.9**	11.2–22.2	**13.0**	9.3–17.2	**30.7**	23.2–39.2
Nationality								
Foreign	**1.0**	0.1–2.1	**67.5**	59.9–76.1	**2.7**	1.1–4.4	**0.0**	-
Education								
Less than primary	**10.5**	6.3–15.0	**10.2**	5.7–15.2	**47.6**	40.8–54.2	**79.0**	67.4–87.7
Primary completed	**68.7**	61.4–75.7	**69.3**	62.3–76.0	**41.1**	34.8–47.3	**16.7**	8.1–27.8
Secondary completed	**20.8**	14.9–26.8	**20.4**	15.3–25.8	**11.3**	7.2–16.5	**4.3**	2.3–7.0
Years living in current residence								
<3 years	**39.8**	32.4–47.4	**55.0**	47.4–62.0	**56.6**	49.9–63.2	**11.6**	7.0–17.5
> = 3 years	**60.2**	52.6–67.6	**45.0**	38.0–52.6	**43.4**	36.8–50.1	**88.4**	82.5–93.0
Was away from residence								
In the past year	**56.5**	48.8–63.3	**27.4**	21.6–33.8	**48.2**	41.5–55.1	**8.5**	5.1–13.2
Present relationship								
Married/ cohabiting	**28.7**	22.2–35.4	**8.2**	2.9–15.1	**1.2**	0.3–2.3	**54.1**	44.0–6.3
Single, never married/ cohabiting	**70.5**	63.6–77.1	**31.0**	24.1–37.5	**61.8**	55.1–67.7	**3.5**	1.2–6.8
Single, previously married/ cohabiting	**0.8**	0.2–1.6	**60.8**	52.9–68.8	**37.1**	31.1–43.7	**42.4**	33.4–52.6
No of commercial sex acts in the past month								
< = 15	**30.6**	23.3–37.9	**15.0**	10.6–20.2	**8.8**	5.7–12.2	**41.9**	31.8–51.7
16–25	**25.0**	18.8–31.4	**26.0**	18.3–33.0	**73.3**	67.6–78.4	**55.6**	45.8–65.5
26–40	**20.9**	15.2–27.1	**32.2**	24.5–40.6	**17.6**	13.1–22.4	**2.5**	0.8–4.6
>40	**23.5**	18.0–29.2	**26.7**	20.2–33.2	**0.3**	0.2–0.8		
Non-commercial sex partners in the past month								
Regular partner*	**46.8**	39.6–54.2	**33.8**	26.0–41.0	**51.7**	44.9–58.3	**96.8**	94.2–98.8
Occasional partner*	**20.2**	14.7–25.9	**48.7**	40.9–56.5	**24.0**	17.7–30.7	**59.6**	50.0–69.4
Has other source of income								
Yes	**10.5**	6.5–15.0	**19.2**	13.9–25.1	**42.6**	36.3–49.0	**27.8**	21.2–35.1

*A ‘regular’ partner was defined as ‘a long-standing non-commercial partner who did not give money or gifts in return for sex and towards whom the sex workers feels an emotional attachment’ and an occasional partner as ‘those partners other than regular partner(s) who did not give you money or gifts in return for sex’.

### Place of care

Table 2 presents the results of where FSWs usually procure male condoms, seek general health care and where they sought care for different HIV and SRH services, the last time they used the service, and the results of the pairwise comparison between cities. In Durban, public health facilities were the most important source for condoms (65%) followed by shops or supermarkets (29%), community workers (26%) and entertainment venues (25%). FSWs in Tete most commonly cited the Night Clinic (37%), on the street/market (31%) and public health facilities (23%). In Mombasa, FSWs frequently procured condoms at public health facilities (42%), pharmacies (33%), and shops or supermarkets (28%). In Mysore, the most common place was from the Ashodaya clinic and Ashodaya organisation (100%). More than one third reported that they get them from friends (36%), 24% at public health facilities and 19% from peer educators.

**Table 2:** Place where FSWs seek care for different HIV/SRH services, by city.

	Durban	Tete	Mombasa	Mysore	Tete vs Durban	Mombasa vs Durban	Mysore vs Durban	Mombasa vs Tete	Mysore vs Tete	Mysore vs Mombasa
	RDS-Adjusted %				Pairwise comparison: Odds Ratio (95% CI)					
Place where male condoms are normally obtained**										
	N = 399	N = 308	N = 400	N = 458						
Public health facility	**64.7**	**22.8**	**42.8**	**24.0**	0.15 (0.08–0.30)	0.41 (0.24–0.70)	0.17 (0.09–0.33)	2.72 (1.41–5.25)	1.15 (0.55–2.42)	0.42 (0.23–0.77)
Targeted clinics	**-**	**36.0**	**-**	**100.0**	-	-	-	-	-	-
Pharmacy/ Chemist	**8.9**	**5.8**	**33.3**	**0.9**	0.63 (0.11–3.75)	5.02 (2.22–11.3)	0.10 (0.02–0.56)	7.91 (1.48–42.2)	0.15 (0.02–1.49)	0.02 (0.00–0.10)
Shop/Supermarket/Petrol station	**29.1**	**5.5**	**27.6**	**1.3**	0.14 (0.05–0.37)	0.90 (0.50–1.64)	0.04 (0.01–0.25)	6.38 (2.59–15.7)	0.27 (0.04–1.97)	0.04 (0.01–0.27)
Café/Bar/Night club/Hotel	**24.9**	**1.9**	**13.3**	**0.0**	0.09 (0.01–0.62)	0.47 (0.24–0.94)	-	5.12 (0.74–35.2)	-	-
Market/Stand/Street vendor	**1.2**	**30.9**	**1.4**	**1.4**	37.6 (5.51–256)	1.29 (0.14–11.5)	1.05 (0.01–217)	0.03 (0.01–0.11)	0.03 (0.00–4.18)	0.81 (0.00–135)
Peer Educators/ CHW	**25.7**	**11.6**	**9.0**	**19.1**	0.35 (0.17–0.69)	0.29 (0.14–0.56)	0.66 (0.31–1.41)	0.82 (0.39–1.74)	1.89 (0.82–4.33)	2.30 (1.01–5.22)
Organisations	**13.8**	**12.6**	**6.4**	**100.0**	0.79 (0.40–1.55)	0.50 (0.26–0.99)	-	0.64 (0.30–1.35)	-	-
Friends	**6.9**	**2.6**	**2.7**	**35.6**	0.52 (0.06–4.31)	0.38 (0.08–1.79)	7.33 (1.96–27.4)	0.74 (0.10–5.27)	14.2 (2.36–85.0)	19.2 (6.73–54.5)
Place where general health care is normally sought										
	N = 400	N = 275	N = 400	N = 458						
Public health facility	**89.1**	**78.0**	**78.6**	**75.0**	0.41 (0.18–0.92)	0.39 (0.19–0.83)	0.39 (0.17–0.93)	0.96 (0.52–1.78)	0.96 (0.46–2.02)	0.98 (0.51–1.96)
Private health facility	**2.1**	**1.8**	**17.4**	**16.3**	1.21 (0.14–10.6)	8.94 (2.44–32.8)	7.08 (1.71–29.4)	1.21 (0.14–10.6)	8.94 (2.44–32.8)	7.08 (1.71–29.4)
Targeted services	**9.0**	**16.7**	**1.5**	**25.8**	1.94 (0.83–4.55)	0.16 (0.05–0.55)	3.06 (1.09–8.64)	1.94 (0.83–4.55)	0.16 (0.05–0.55)	3.06 (1.09–8.64)
Pharmacy/ Chemist	**3.5**	**6.0**	**2.5**	**0.0**	1.70 (0.50–5.83)	0.81 (0.22–2.94)	0.19 (0.00–19.2)	1.70 (0.50–5.83)	0.81 (0.22–2.94)	0.19 (0.00–19.2)
Traditional healer	**0.1**	**0.0**	(0.2)*	(0.2)*	-	-	-	-	-	-
Place where contraception was last obtained^1^										
	N = 131	N = 177	N = 247	N = 351						
Public health facility	**88.3**	**36.4**	**56.0**	**96.3**	0.08 (0.02–0.26)	0.15 (0.05–0.47)	3.07 (1.67–0.74)	1.92 (0.91–4.04)	38.6 (12.1–123)	20.1 (6.78–59.8)
Private health facility	**2.0**	(0.9)*	**21.4**	**3.2**	0.46 (0.01–23.2)	14.2 (0.27–744)	1.90 (0.03–110)	31.1 (17.2–56.5)	4.15 (1.35–12.8)	0.13 (0.04–0.45)
Targeted services	**8.3**	**32.0**	**5.4**	**0.5**	4.91 (1.12–21.6)	0.79 (0.17–3.62)	0.05 (0.00–1.68)	0.16 (0.06–0.43)	0.01 (0.00–0.28)	0.06 (0.00–1.79)
Pharmacy/ Chemist	**0.0**	**11.8**	**6.8**	**0.0**	-	-	-	0.66 (0.21–2.15)	-	-
Place originally from	**-**	**13.6**	(1.8)*	**-**	-	-	-	0.13 (0.02–0.85)	-	-
Other	**1.4**	**6.3**	**2.6**	**0.0**	2.84 (0.42–18.9)	6.38 (1.06–38.3)	-	2.25 (0.44–11.4)	-	-
Place where care sought for last STI/RTI syndrome^2^										
	N = 174	N = 132	N = 64	N = 101						
Public health facility	**84.2**	**60.4**	**54.6**	(12.0)*	0.30 (0.10–0.86)	0.21 (0.06–0.71)	0.02 (0.00–0.15)	0.69 (0.23–2.02)	0.06 (0.01–0.44)	0.08 (0.01–0.72)
Private health facility	**1.7**	**0.0**	**17.3**	**1.7**	-	14.9 (0.49–447)	1.03 (0.02–61.5)	-	-	0.07 (0.00–1.02)
Targeted services	**3.9**	**24.1**	(5.4)*	**89.4**	4.91 (0.81–30.0)	1.41 (0.16–12.2)	148 (15.5–1414)	0.29 (0.06–1.29)	30.2 (5.90–154)	105 (14.0–790)
Pharmacy/ Chemist	**6.3**	**4.4**	(6.4)*	(0.6)*	0.58 (0.09–3.90)	0.92 (0.10–8.46)	0.08 (0.02–0.35)	1.58 (0.18–14.3)	0.15 (0.04–0.59)	0.09 (0.02–0.56)
Place originally from	**-**	**7.9**	**14.3**	**-**	-	-	-	1.30 (0.07–24.5)	-	-
Place where last tested for HIV^3^										
	N = 204	N = 241	N = 326	N = 414						
VCT centre	**7.8**	**4.4**	(1.4)*	**16.9**	0.52 (0.03–2.09)	0.15 (0.00–5.34)	2.29 (0.45–11.7)	0.29 (0.01–10.5)	4.37 (0.81–23.7)	14.9 (0.38–586)
Public health facility	**49.6**	**39.1**	**48.5**	**3.4**	0.69 (0.33–1.44)	0.91 (0.47–1.76)	0.04 (0.01–0.10)	1.31 (0.71–2.45)	0.05 (0.02–0.14)	0.04 (0.02–0.10)
Private health facility	**2.4**	(0.6)*	**17.0**	**1.0**	0.28 (0.00–248)	8.84 (0.02–3606)	0.54 (0.00–293)	31.4 (1.24–798)	1.91 (0.04–81.3)	0.06 (0.01–0.47)
Targeted services	**29.2**	**16.0**	**10.6**	**78.6**	0.45 (0.16–1.21)	0.31 (0.13–0.71)	8.96 (2.72–29.5)	0.69 (0.26–1.82)	20.1 (5.54–73.0)	29.0 (9.08–92.6)
Youth-friendly services	**10.1**	**0.0**	(0.1)*	**0.0**	-	0.01 (0.00–0.02)	-	-	-	-
Community VCT	**0.0**	**21.6**	**9.2**	**0.0**	-	-	-	0.42 (0.19–0.93)	-	-
Place where using HIV care services^4^										
	N = 38	N = 104	N = 32	N = 29						
Public health facility	**96.5**	**61.0**	(50.0)*	**100.0**	0.03 (0.00–0.63)	0.03 (0.00–0.71)	-	0.97 (0.25–3.70)	-	-
Private health facility	**0.6**	**0.0**	(42.7)*	**0.0**	-	-	-	-	-	-
Place originally from	**-**	**38.9**	(5.1)*	**-**	-	-	-	0.04 (0.02–0.10)	-	-
Place where last tested for cervical cancer^5^										
	N = 110	-	N = 49	N = 50						
Public health facility	**96.9**	**-**	(36.9)*	(48.9)*	-	0.02 (0.00–0.09)	-	0.03 (0.01–0.16)	-	1.64 (0.36–7.41)
Private health facility	(0.8)*	**-**	(19.5)*	(2.9)*	-	-	-	-	-	-
Targeted services	**0.0**	**-**	(38.8)*	(40.0)*	-	-	-	-	-	-

* Bootstrap analysis was not possible because of too few observations in some categories. A weighed proportion was calculated instead.

**Multiple answers possible

^1^ N = Using a non-barrier FP method

^2^ N = Had STI/RTI syndrome in past year and sought care

^3^ N = Tested for HIV less than 3 years ago

^4^ N = Currently using pre-ART or ART services

^5^ N = Was ever tested for cervical cancer

General health care, such as when ill, at all cities was most commonly sought at public health facilities. Private facilities were also an important source in Mombasa (17%) and Mysore (16%), and in Tete and Mysore the clinics specifically targeting FSWs (respectively 17% and 26%). Public health facilities were also the most often source for contraceptive services in all cities, although in Tete a similar number sought it at the Night Clinic (32%) and in Mombasa 21% got them from private clinics.

In the three African cities, public health facilities were the most common place where FSWs received care for their last STI complaint (84% in Durban, 60% in Tete and 55% in Mombasa), but this was not the case in Mysore where the large majority attended these services at the SW-specific Ashodaya clinic (89%). In Tete, a quarter sought it at the Night Clinic and in Mombasa 17% at private clinics. A similar picture was observed for HTS with the public health facilities being the most important place in Durban (50%), Tete (39%) and Mombasa (49%), while in Mysore 79% last tested at the Ashodaya clinic. In the African cities, 29%, 16% and 11% had been tested by HTS specifically targeting FSWs, respectively in Durban, Tete and Mombasa. In Durban, outreach HTS by a non-governmental agency specifically targeting youth (< = 35 years) appeared to be another important occasion for FSWs to be tested (10%). In Tete many had been tested by (government) community outreach (22%) or in their place of origin (18%), and in Mombasa private clinics were reported by 17% of FSWs. In Mysore, the only other place of importance were public HTS centres (17%).

FSWs who were receiving HIV care, either pre-ART or ART, were all or almost all in care at public health facilities in Durban (96%) and Mysore (100%). Also in Tete, most were followed at local public health facilities (61%) and the remaining at their place of origin. Only in Mombasa, a substantial proportion (43%) reported to be in care in the private sector. In Durban, almost all FSWs had been screened for cervical cancer in the public sector (97%), while in Mombasa and Mysore a substantial proportion (respectively 39% and 40%) was screened at the clinics specifically targeting FSWs.

### Reasons for choice of place of care

Table 3 presents the results of the multivariate analysis of the reasons why a place was chosen, for all HIV/SRH visits combined. FSWs in Durban responded by far most frequently because it was nearby (61.5%), followed by because of the quality of care (23.0%) and the cost (17.4%). In Tete, the most common answers were ‘because that is where I always go’ (60.1%) and because it was nearby (41.7%). In Mombasa, the most common responses were because it was nearby (47.5%), the cost (43.2%), ‘because it is where I always go’ (35.3%) and the quality of care (25.9%). In Mysore, cost (76.9%) and ‘where I always go’ were the most common reasons, followed by privacy (55.9%) and being nearby (34.3%).

**Table 3:** Reasons for choice of place of care last time care was sought, for all SRH visits combined, by city*.

	Durban N = 542	Tete N = 653	Mombasa N = 682	Mysore N = 911	Tete vs Durban	Mombasa vs Durban	Mysore vs Durban	Mombasa vs Tete	Mysore vs Tete	Mysore vs Mombasa
	RDS-Adjusted %				Pairwise comparison: Odds Ratio (95% CI)					
Cost is low or free	**17.4**	**3.3**	**43.2**	**76.9**	0.18 (0.08–0.44)	4.63 (2.50–8.54)	14.8 (6.96–31.5)	24.4 (11.5–52.1)	78.3 (32.4–189)	3.20 (1.69–6.09)
Good value for money	**0.8**	**-**	**1.0**	**8.3**	-	0.68 (0.03–14.2)	6.50 (0.15–291)	-	-	9.57 (0.42–220)
Shorter waiting times	**2.9**	**6.7**	**3.9**	**20.9**	2.12 (0.70–6.41)	1.17 (0.37–3.74)	4.64 (1.42–15.1)	0.56 (0.20–1.51)	2.19 (0.91–5.25)	3.94 (1.24–12.5)
Nearby	**61.5**	**41.7**	**47.5**	**34.3**	0.70 (0.40–1.23)	0.86 (0.51–1.45)	0.33 (0.18–0.59)	1.23 (0.73–2.07)	0.46 (0.26–0.82)	0.38 (0.21–0.68)
Where I always go	**16.7**	**60.1**	**35.3**	**70.5**	10.4 (5.16–21.0)	3.24 (1.67–6.29)	14.0 (6.84–28.8)	0.31 (0.18–0.53)	1.35 (0.74–2.45)	4.33 (2.43–7.71)
Quality of care	**23.0**	**1.8**	**25.9**	**27.6**	0.04 (0.01–0.13)	1.06 (0.55–2.03)	1.28 (0.57–2.89)	26.9 (8.83–81.8)	32.5 (10.1–104)	1.21 (0.59–2.48)
Privacy	**4.6**	**6.0**	**2.4**	**55.9**	0.99 (0.23–4.27)	0.51 (0.11–2.34)	18.5 (4.06–84.1)	0.51 (0.16–1.66)	18.6 (5.04–68.9)	36.2 (12.4–105)
Friendly health personnel	**3.6**	**4.8**	**2.4**	**27.6**	0.75 (0.16–3.54)	0.47 (0.09–2.51)	3.71 (0.72–19.0)	0.62 (0.23–1.65)	4.93 (1.89–12.8)	7.96 (3.03–20.9)
It was indicated/referred	**4.0**	**22.8**	**5.8**	**5.0**	6.15 (2.29–16.5)	1.55 (0.54–4.45)	1.43 (0.37–5.48)	0.25 (0.13–0.50)	0.23 (0.08–0.70)	0.92 (0.28–3.00)

*Multiple answers possible

The multivariate models comparing reasons for choosing the place of care between care pursued at public, private and targeted services for all cities combined (Table 4), indicated that targeted services were substantially (p<0.005) more often chosen because of the lower cost, the shorter waiting times, quality of care, privacy and the friendly health personnel, compared to public health facilities.

**Table 4:** Pairwise comparison of reasons for choice of place of care between type of provider*,

	RDS-Adjusted %				Pairwise comparison (Odds Ratio and p-value)					
	Public sector (N = 1509)	Private sector (N = 175)	Targeted services (N = 684)	Place of origin (N = 163)	Private vs Public		Targeted vs Public		Targeted vs Private	
	%	%	%	%	OR	95% CI	OR	95% CI	OR	95% CI
Cost is low or free	**31.8**	**24.3**	**40.3**	**11.6**	0.37	0.16–0.87	1.27	0.78–2.06	3.40	1.39–8.30
Good value for money	**2.2**	**2.1**	**2.1**	(4.5)**	2.74	0.47–16.1	0.36	0.15–0.84	0.13	0.02–0.79
Shorter waiting times	**3.5**	**8.7**	**12.9**	**2.1**	6.68	2.11–21.1	2.86	1.42–5.76	0.43	0.11–1.66
Nearby	**54.8**	**37.2**	**47.8**	**18.0**	0.47	0.26–0.88	0.91	0.60–1.38	1.92	0.97–3.82
Where I always go	**40.2**	**40.0**	**58.0**	**62.4**	1.15	0.51–2.61	1.46	0.94–2.26	1.26	0.53–3.00
Quality of care	**15.1**	**28.8**	**29.4**	**14.9**	1.42	0.65–3.11	3.11	1.93–5.02	2.19	0.93–5.18
Privacy	**6.1**	**3.8**	**31.9**	**3.6**	2.63	0.75–9.25	5.83	3.12–10.9	2.21	0.60–8.11
Friendly health personnel	**3.6**	**5.6**	**14.0**	**2.8**	2.02	0.66–6.23	2.59	1.23–5.47	1.28	0.39–4.26
It was indicated/referred	**10.2**	**6.7**	**11.8**	**4.2**	1.24	0.37–4.11	0.94	0.49–1.82	0.76	0.20–2.85

* N = all HIV/SRH visits at public, private and targeted services, multiple answers possible

** Bootstrap analysis was not possible because of too few observations in some categories. A weighed proportion was calculated instead.

## Discussion

We assessed SRH care seeking practices in four cities with different health service delivery options for sex workers. Such a standardised comparison across cities has to our knowledge never been done and it clearly shows that where and why FSWs seek care is highly context-specific.

At the time of the survey, there was a broad range of public and private health facilities in Durban, but no facilities that specifically targeted FSWs or key populations. The only targeted service was mobile outreach by a non-governmental agency that mostly focused on condom distribution and HIV testing. It is therefore not surprising that only these two commodities were reported by a substantive proportion of FSWs to have been received from targeted services. The same non-governmental agency also had a youth outreach programme, which in South Africa is broadly defined as anyone up to 35 years, and an important number of FSWs, of which most are 35 years or less, had been reached by this programme. Nevertheless, also for condoms and HTS the public health facilities were a more important source of care.

In the Tete-Moatize area, there is a small stand-alone clinic at the outskirts of Moatize offering condoms, contraceptive services, STI care and HTS during the evening. The clinic was a prominent source of condoms, contraception and STI care, with about one third of FSWs reporting it, and less for HTS, with about one sixth reporting it. The public sector was, however, also here the most important service provider. A particular characteristic of the Tete FSW population is that a large proportion of the FSWs did not seek care in the Tete-Moatize area, but at their place of origin. These are mostly FSWs of Zimbabwean origin, who comprised two thirds of the FSW population. This is an important fact to take into account when implementing interventions to improve access to SRH services. In particular for services requiring ongoing care and repeated visits, such as HIV care and contraception, it has to be explored whether services are best procured locally or at the place of origin and how linkage between both might be improved. The link between mobility and poor retention in HIV care has been well documented but effective strategies to tackle the problem are still lacking [5].

In Mombasa, three drop-in clinics, in different locations across town, operate specifically for FSWs and offer condoms, contraception, STI care, HTS and cervical cancer screening. In addition, FSW-targeted services are offered, in the context of ongoing research, at a clinic in Mombasa [15] and another in neighbouring Kilifi [20]. The coverage of these clinics was modest, however, with not more than 10% of the FSWs reporting it as place of care for most services. Only for cervical cancer screening it was important, with more than one third screened, possibly because cervical cancer screening is still not widely available at the general health facilities in Mombasa [21]. Mombasa was the only site where a relatively large proportion of FSWs received care in the private-for-profit sector. In none of the other sites was this an important source of care. The probable reason is that the Kenya private sector is one of the most developed and dynamic in sub-Saharan Africa [22]. In the focus group discussions that were concurrently held with FSWs in Mombasa, those who preferred private clinics mentioned as main reasons that they are treated well and with respect at these clinics, and that stigma and discrimination were minimal [21].

Mysore is characterised by the large presence of the Ashodaya clinic. This clinic was established in 2004 and is operated by a sex worker collective. It provides STI/HIV/AIDS prevention services specifically for sex workers and has become by far the most important STI care and HTS provider for this population, as well as a key provider of cervical cancer screening [16]. For services not offered at the clinic, such as contraception, the public sector remains the most important provider. In this context, it might be worth expanding the set of services at the clinic to include contraception.

Also the reasons for seeking care at a specific place differed by city. Cost appears particularly important in Mysore and, to a smaller degree, in Mombasa, but less so in Durban and Tete. A possible explanation is that in these two latter cities, HIV/SRH services have always been free at public health facilities and users have gotten used to it. Proximity appears to be an important criterion at the African sites and less in Mysore. Many FSWs also reported that it is ‘where they always go’ as the reason, indicating that FSWs might often choose a health facility for all their medical care needs and become accustomed to navigate services at the facility, rather than choosing different types of facilities for different needs. It also might signal that newly introduced services may initially have low uptake as FSWs may be reluctant to move from existing services.

Though FSWs gave mostly practical reasons for choosing services, such as habit, proximity or cost, motives related to quality of care were relatively more highlighted by users of targeted services, which could indicate that they perceive these specialised services to have a higher quality of care than other providers. This is consistent with what has been documented elsewhere [1, 2, 11].

There is no standard model of how best to ensure access to HIV/SRH services for FSWs [10]. Stand-alone clinics specific for FSWs, such as the Ashodaya clinic, the Night Clinic and the DICs, can offer services tailored to FSWs’ needs in a non-stigmatising environment and during appropriate hours, and have shown to have a positive effect on health seeking behaviours in several settings [13, 23, 24]. They are, however, often not endorsed or favoured by governments and most commonly require additional funding, often in the form of project funding, which may not be sustainable [23–27]. A review of facility-based SRH services for female sex workers in Africa concluded that targeted services have limited coverage and a narrow scope of services, mostly focusing on HIV and STI interventions rather than on broader SRH services [26]. A less expensive and more sustainable alternative may be to reduce the barriers in accessing the regular services, such as a negative reception by providers and other users. These barriers are however often difficult to alleviate and lower service coverage is generally achieved than by stand-alone FSW clinics [28]. Our baseline assessment shows that targeted services have the potential to reach a large proportion of FSWs, as is the case for STI care and HTS in Mysore, but that their presence does not guarantee high coverage, as is observed in Mombasa and Tete.

The face-to-face interviews we conducted provided valuable quantitative information on where and why FSWs seek care, but this method faces limitations such as recollection bias, poor understanding of the question or social desirability bias. In Tete an electronic questionnaire was used, while in the other cities it was paper-based, but we do not believe that this has substantially influenced the comparability of the results. Reporting bias has never been shown to be substantially different between face-to-face electronic and paper-based questionnaires, unlike between face-to-face and self-administered questionnaires [29, 30].

In a next phase, the results were triangulated with the results of the focus group discussions, key informant interviews and health facility assessments that were conducted concurrently [21] and a city-specific intervention package was developed. The DIFFER project aims at using a ‘diagonal approach’ whereby it is assessed what services are best provided to FSWs targeted (vertical), what services are best provided by the regular health system (horizontal) and how the linkage between both can be improved. The baseline assessment shows that this needs to be site-specific, taking into account the current coverage by targeted and regular health services and the reasons why a place is chosen.

## Conclusion

Current care seeking for different HIV/SRH services and commodities, and the reach of FSW-targeted services, differs substantially between study cities. Reasons for choosing a particular place of care are mostly practical, such as being nearby. Health services specifically targeted at FSWs are relatively more often chosen because of their perceived higher quality of care. The best model to improve access to care needs to be tailored to the specific context of each city. It should combine the strengthening and expanding of targeted services where relevant and sustainable, with improving access to and linkages with the general health services.

## Supporting Information

S1 Dataset Survey dataset. (DTA)
